# Follicular-Stimulating Hormone, Luteinizing Hormone, and Prolactin Serum Level in Patients with Oral Lichen Planus in Comparison to Healthy Population

**DOI:** 10.1155/2021/8679505

**Published:** 2021-10-26

**Authors:** Fatemeh Lavaee, Nazila Bazrafkan, Fateme Zarei, Maryam Shahrokhi Sardo

**Affiliations:** ^1^Oral and Dental Disease Research Center, Oral and Maxillofacial Medicine Department, School of Dentistry, Shiraz University of Medical Sciences, Shiraz, Iran; ^2^Student Research Committee, Shiraz University of Medical Sciences, Shiraz, Iran

## Abstract

**Materials and Methods:**

In this cross-sectional case control study, the serum level of LH, FSH, and prolactin of 40 women with lichen planus who have been referred to Shiraz Dental Faculty, Oral and Maxillofacial Disease Department during 2018-2019 has been evaluated in comparison to 40 healthy controls. Data were analyzed by SPSS version 18. Two-way ANOVA and Mann–Whitney test were used for data analysis.

**Results:**

The mean serum level of FSH and LH was significantly higher in OLP patients while this difference was not reported for prolactin. Only FSH mean serum level was significantly higher in nonmenopausal OLP patients. The distribution of prolactin and FSH hormones' serum level was in normal range.

**Conclusions:**

The high serum level of FSH and LH can affect OLP pathogenesis by estrogen and progesterone modulation.

## 1. Introduction

Oral lichen planus (OLP) is an oral potentially malignant disorder, with a frequency of prevalence of 1.4% in the world. Oral lichen planus is a T cell-mediated autoimmune disease with an unknown etiology, but some evidences showed that cytotoxic CD8+ cells induce oral epithelial cell apoptosis. Also, cytotoxic CD8+ cells affect the keratinocytes with specific surface antigens and recognized the basal epithelial cells as foreign body which lead to basal epithelial layer degeneration [1–3]. OLP has slight prevalence in women during 30-60 years old [3–5]. There are some reports about the susceptibility of women for autoimmune diseases [6, 7].

Women are more prone for these diseases such as systemic lupus erythematous Hashimoto thyroiditis. The role of sex hormones in triggering the immune system has been shown in previous studies [6–8].

Higher incidence of autoimmune disorders in women may be indicative of the effects of sex hormones. Sex hormones may affect the autoimmune diseases pathogenesis [6, 7].

Prolonged estrogenic stimulation which is caused by abnormal estrogen metabolism has been reported in patients with SLE [9]. Pregnancy and menarche hormonal changes are shown to affect several autoimmune diseases, such as SLE, autoimmune thyroiditis, Graves' disease, and rheumatoid arthritis [10, 11]. For example, in one study, progesterone level has been shown to be lower in SLE patients [12].

SLE may worsen during pregnancy, while other autoimmune diseases like MS and RA are improved during this period [13].

Since OLP is an autoimmune disease and also there is no evaluation in literature about the relation of sexual hormone on clinical aspect of OLP presentation, in this study, we aimed to evaluate any possible relation of FSH, LH, and prolactin level and OLP.

## 2. Materials and Methods

This cross-sectional case-control study was done on women with OLP who have been referred to Shiraz Dental Faculty, Oral and Maxillofacial Disease Department during 2018-2019. This study was conducted according to the ethical principles of Helsinki (version 2002), and it was approved by the ethics committee of Shiraz University of Medical Sciences (IR.SUMS.REC.1397.735).

The international diagnostic criteria for oral lichen planus have been considered. Bilateral reticular steria, white plaque, annular or plaque-like lesions with radiating lines, atrophic or erosive, or bollouse lesions are different clinical manifestation of OLP. OLP histopathological findings are liquefaction degeneration of the basal epithelial layer, band-like infiltration of lymphocytes, and civatte bodies in connective tissue [14].

Patients with histopathologically confirmed lichen planus who were admitted or treated outpatient were enrolled in this study. Pregnant, lactating women, and active hormone therapy during study were excluded (steroid therapy, exogenous administration of other evaluated hormone, each medication which can affect sex hormonal profile). Also, the patients with other autoimmune diseases or any inflammatory disorders and malignancies had been excluded. Since there was no similar study for sample size assessment, the results of the most similar study about sjogren as an autoimmune disease had been considered for sample size calculation.

With power of 80%, the type 1 error is of 0.05. Considering the result of evaluation about the relation of sex hormone and correlation coefficient of 0.58, the sample size has been estimated at least 21 participants. 40 participants have been enrolled in each group [15].

In the control group, healthy volunteer women who met the above exclusion criteria and were age matched with our participants in the OLP group were enrolled in the study. Forty women were recruited in each group. Their serum level of sex hormones was evaluated if they had signed the written consent form. The blood samples were obtained during their routine evaluation before starting corticosteroid therapy on day 3 of menstruation period and 2 hours after their morning awaking, and this specific day of blood sample obtaining was not necessary for menopausal patients.

After blood sample collecting, they were balanced and after 15-20 minutes, the samples were centrifuged by 3500 rpm for 7 minutes. Serum samples that did not intend to be tested on the same day were frozen. The serum level of LH, FSH, and prolactin was evaluated in Motahari laboratory by the CLA method. LH, FSH, and prolactin have been assessed by Gama kit, testosterone by Autobio, DHEA-s by Elisa kit, and progesterone and estradiol by Zimense kit (made in Germany).

Patients' demographic data including age, severity of their OLP lesions (erosive and nonerosive OLP), and menopausal condition were registered. Data were analyzed by SPSS version 18. The normality of data has been assessed by Kolmogorov-Smirnov test. Mann–Whitney test was used for comparing the hormonal mean of OLP and healthy control groups and also comparing these hormones between different severities of OLP lesion. *P* value less than 0.05 was considered significant.

## 3. Results

In this study, 40 participants had been enrolled in both healthy control and OLP groups. The normality of data has been assessed by Kolmogorov-Smirnov test. The data distribution was not normal for all evaluated hormones.

The mean age of patients with OLP and healthy control groups was 51.9 ± 11.52 and 49.38 ± 11.65 years old, respectively (menopause participant mean age 35.5 ± 11.13 and nonmenopause participant mean age 51.23 ± 11.61 years old). Since the age of participants in OLP and healthy control groups had been matched, there was no significant difference between the age of both groups (*P* value = 0.33).

The number of menopause and nonmenopause participants in the OLP group was not statistically different from the healthy controls (*P* value = 0.072).

The mean serum level and comparison of evaluated hormones between menopause participants of OLP and healthy control groups, which is presented in Table 1, were not significantly different.

The serum level of hormones (except FSH) in nonmenopause participants of the OLP group was not different from healthy control (Table 2).

The menopause participants with OLP had significantly different serum level of prolactin, FSH, and LH in comparison to the nonmenopause ones. The serum level values are reported in Table 3.

In the control group, the mean serum level FSH and LH in menopause participants was significantly higher than nonmenopause ones (Table 4).

In this evaluation, 29 patients have erosive form of OLP while 11 patients have nonerosive form (keratotic).

The comparison of mean value of FSH, LH, and prolactin in OLP patients with erosive and nonerosive form is represented in Table 5. Type of OLP and its severity did not affect the serum level of prolactin (*P* value = 0.39), FSH (*P* value = 0.47), and LH (*P* value = 0.57).

The distribution of hormones either normal or abnormal range is reported in Table 6. The standard hormone ranges for menopause and nonmenopause participants and their phase of blood sampling in menopause participants (follicular phase in our study) have been considered according to the standard of kit used by laboratory as a point for determining the normal or abnormal distribution.

The number OLP patients with abnormal range of LH serum level was as high as normal range (*P* value >0.999).

The number of patients with normal and abnormal range of hormones is compared between OLP patients and healthy controls.

## 4. Discussion

According to the results of this study, the mean serum level of FSH and LH was significantly higher in OLP patients while this difference was not reported for prolactin.

Considering the menopausal status of participants, only FSH mean serum level was significantly higher in nonmenopausal OLP patients. In contrary to what is expected for the effect of menopausal status on prolactin serum level, in OLP patients, the mean serum level of prolactin was significantly higher in nonmenopause participants, while this value was higher in menopause participants of the healthy control group. The distribution of FSH and prolactin hormones' serum level were in normal range.

There are some studies in literature in which the relation of sex hormones and autoimmune diseases has been assessed.

In a study on 39 patients with OLP and 39 healthy controls from both gender and age range of 15-55 years old, there was no difference between prolactin serum level between two OLP patients and healthy controls (*P* value = 0.28). A significant difference between the mean of prolactin in the OLP group before and after treatment was noted (*P* value <0.001). Also, there was no correlation between OLP duration and mean serum prolactin levels. In both remission and exacerbation phase of OLP, no difference has been reported between men and women (*P* value >0.05). Prolactin decreased significantly in remission phase of OLP.

Although the findings of this study confirm our results about prolactin, but the design of these two studies is different. Comparing both men and women in a group is not proper, since the serum level of these hormones varies in men and women; also, the menopause status can affect serum level of sex-related hormones [16].

Ghalaut et al. reported higher level of prolactin in OLP patients in comparison to healthy controls, while in the present study, the prolactin serum level in OLP patients was not significantly different (*P* value = 0.31) from healthy controls [17].

OLP as one of autoimmune diseases with clinical presentation of desquamative gingivitis has been assessed for estrogen receptors in desquamated gingiva. This evaluation revealed no correlation between the diseased and estrogen receptors' presence. This finding cannot support estrogen therapy for autoimmune or idiopathic origin of desquamative gingivitis [18].

A study has reported alleviating effect of decreased level of prolactin in animal models of autoimmune diseases [19].

Psoriasis is an autoimmune disease with similar pathogenesis to OLP. Several evaluations confirmed the higher level of prolactin in patients with psoriasis [20–22]; also, they reported the therapeutic effects of bromocriptine (antiprolactin secretion) on different autoimmune diseases including psoriasis, rheumatoid arthritis, and Reiter's syndrome [19].

However, few studies did not show significant difference between serum level of prolactin in healthy controls and patients with psoriasis [23, 24]. This result is confirmed by presenting assessment on OLP patients.

Prolactin secretion can be affected by smoking, antipsychotic drugs, and emotional stress. Also, different autoimmune diseases may have different pathogenesis. Studies are conducted in different phases of diseases, and genetic, ethnic, cultural and environmental, and psychotic confiders can affect the results. It can be suggested to consider and homogenize these factors.

Also, most of these controversies are related to the study design and sex and menopause situation of enrolled participants which have not been considered preciously in many previous studies. Enrolling both men and women, menopause participants, and nonmenopause participants together in evaluation group are a great confounding factor. In our study, we considered these items.

Prolactin like growth hormone belongs to the pituitary polypeptide hormone family with a common structure with growth hormone [25]. Prolactin can modulate cell growth and proliferate human keratinocytes [25, 26]. Prolactin is immune stimulator and promotes autoimmunity by increasing IL-2, IFN-gamma, and autoantibody by regulating Th_1_ and Th_2_ lymphocytes, respectively [27]. These T cells are playing an important role ion pathogenesis of OLP. Dense subepithelial lymphocyte infiltration in addition to intraepithelial lymphocyte and basal keratinocyte degeneration is the well-known pathologic aspects of OLP [28]. CD8^+^ T cells are the most dominant cells and activate other T cells and damage basal keratinocytes [28–30]. According to these relations between the prolactin mechanism of action and OLP pathogenesis, it can be assumed that prolactin can modulate OLP pathogenesis.

In spite of what we have reported for prolactin, mean serum levels of FSH and LH have been significantly higher in patients with OLP. These gonadotropin hormones modulate estrogen and progesterone secretion, respectively. High level of these hormones can be related to the gonadal hypofunction, so the FSH and LH role in pathogenesis of OLP or other autoimmune disease is indirectly and through gonadal hormones including estrogen, progesterone, and androgens.

OLP is more prevalent in premenopausal women which is associated with gonadotropin estrogen and progesterone fluctuations [31]. FSH and LH increment in menopausal status is due to feedback cycle of estrogen and progesterone reduction [32, 33]. These hormonal changes may participate in etiopathogenesis of OLP. While estrogen reinforced the humoral immune response, its effect on cellular immunity, which plays an important role in OLP pathogenesis, is different.

Estrogen regulates CD_4_^+^ (Th_1_, Th_2_, Th_17_, and Tregs) and CD_8_^+^ cells. Tregs downregulate the immunologic reactions. These T cells are increased by estrogen. So the immunologic protective effective of estrogen can be justified [34]. Estrogen can regulate INF-*γ*, TNF-*α*, IL-6, and IL-13 [35–37].

Previous studies have controversial reports, while some studies confirmed the alleviating effect of estrogen, estriol, and estradiol on autoimmune diseases such as multiple sclerosis [38]; other evaluations did not show significant symptom improvement for autoimmune diseases such as rheumatoid arthritis (RA) [39]. However, antiprolactin and estrogen medications have been suggested for SLE in another evaluation [40, 41].

In the presenting study, the serum level of FSH gonadotropin hormone was significantly higher which can be associated with lower level of estrogen. This protective effect of estrogen is confirmed by other studies [38]. In accordance to what we found about FSH, LH was also significantly higher in OLP patients. This is associated logically with lower level of progesterone. This hypothesis is confirmed by Hughes and Choubey in SLE patients. They concluded that progesterone can reduce the risk of SLE [41].

Progesterone is a natural immune suppressor which suppresses CD_4_^+^ T cell proliferation and differentiation, enhances. It increases IL-4 secretion and Treg cell differentiation and reduces INF-*γ*,TH_17_ responses, and T cell-dependent antibody response, which can suppress immune response [34].

Overall higher serum of FSH and LH in OLP patients, which can be indicative of lower level of estrogen and progesterone, may be related to OLP pathogenesis; so more precise assessment on these hormonal fluctuations in different period is needed to describe it better.

In this study, the OLP patients who were not under steroid therapy had been enrolled in this evaluation. Since systemic steroid therapy, even long term topical steroid therapy can cause adrenal suppression.

It can be suggested to consider and assess all effective factors including BMI and environmental situations. Since sex hormone can be affected by BMI, sexual steroid hormones can play a causative role for metabolic disorders and obesity [42].

## 5. Conclusions

According to the results of this study, the mean serum level of FSH and LH was significantly higher in OLP patients while this difference was not reported for prolactin.

FSH and LH fluctuation can affect OLP pathogenesis by estrogen and progesterone modulation.

Considering the menopausal status of participants, only FSH mean serum level was significantly higher in nonmenopausal OLP patients.

## Figures and Tables

**Table 1:** The comparison of hormones between menopause participants of OLP and healthy control groups.

Hormones		Number of menopause participants	Mean	Std. deviation	Std. error mean	*P* value
Prolactin (ng/ml)	Patients	27	10.24	6.503	1.252	0.66
	Control	19	15.62	12.365	2.837	
FSH (miu/ml)	Patients	27	78.40	35.675	6.866	0.176
	Control	19	64.78	23.190	5.320	
LH (miu/ml)	Patients	27	43.196	30.37638	5.84594	
	Control	19	28.7579	13.90633	3.19033	0.246

FSH: follicle-stimulating hormone; LH: luteinizing hormone.

**Table 2:** The comparison of hormones between nonmenopause participants of OLP and healthy control groups.

Hormones		Number of nonmenopause participants	Mean	Std. deviation	Std. error mean	*P* value
Prolactin (ng/ml)	Patients	13	20.54	10.802	2.996	
	Control	21	13.95	6.325	1.380	0.07
FSH (miu/ml)	Patients	13	23.08	32.109	8.906	0.003
	Control	21	8.93	11.487	2.507	
LH (miu/ml)	Patients	13	15.0015	25.94848	7.19681	0.156
	Control	21	7.1319	8.62652	1.88246	

FSH: follicle-stimulating hormone; LH: luteinizing hormone.

**Table 3:** The mean serum level and comparison of FSH, LH, and prolactin between menopause and nonmenopause participants of the OLP group.

Hormones in OLP patients		Number	Mean	Std. deviation	Std. error mean	*P* value
Prolactin (ng/ml)	Nonmenopause	13	20.54	10.802	2.996	
	Menopause	27	10.24	6.503	1.252	0.001
FSH (miu/ml)	Nonmenopause	13	23.08	32.109	8.906	<0.001
	Menopause	27	78.40	35.675	6.866	
LH (miu/ml)	Nonmenopause	13	15.0015	25.94848	7.19681	
	Menopause	27	43.1963	30.37638	5.84594	<0.001

FSH: follicle-stimulating hormone; LH: luteinizing hormone.

**Table 4:** The mean serum level and comparison of FSH, LH, and prolactin between menopause and nonmenopause participants of the healthy control group.

Hormones		Number of healthy control group	Mean	Std. deviation	Std. error mean	*P* value
Prolactin (ng/ml)	Nonmenopause	21	13.95	6.325	1.380	
	Menopause	19	15.62	12.365	2.837	0.79
FSH (miu/ml)	Nonmenopause	21	8.93	11.487	2.507	<0.001
	Menopause	19	64.78	23.190	5.320	
LH (miu/ml)	Nonmenopause	21	7.1319	8.62652	1.88246	
	Menopause	19	28.7579	13.90633	3.19033	<0.001

FSH: follicle-stimulating hormone; LH: luteinizing hormone.

**Table 5:** The comparison of mean value of FSH, LH, and prolactin in OLP patients with erosive and nonerosive form.

OLP type	Hormones	Number	Minimum	Maximum	Mean	Std. deviation
Erosive	Prolactin (ng/ml)	29	3.60	36.30	12.6421	7.97642
	FSH (miu/ml)	29	6.06	120.00	63.5486	42.93699
	LH (miu/ml)	29	0.93	110.00	34.1703	31.24307
	Valid N (list wise)	29				
Nonerosive	Prolactin (ng/ml)	11	3.70	44.50	16.0900	12.46812
	FSH (miu/ml)	11	5.28	120.00	52.1891	44.34734
	LH (miu/ml)	11	3.05	97.10	33.6709	34.22388
	Valid N (list wise)	11				

FSH: follicle-stimulating hormone; LH: luteinizing hormone.

**Table 6:** The distribution of hormones either normal or abnormal range.

	LH	FSH	Prolactin
Number of OLP patients			
Normal	20	32	32
Abnormal	20	8	8
*P* value	1	0 < 0.0001	0 < 0.0001
Number of healthy control			
Normal	29	34	30
Abnormal	11	6	10
*P* value	0.004	0 < 0.0001	0.002

FSH: follicle-stimulating hormone; LH: luteinizing hormone.

## Data Availability

The readers can access the data supporting the conclusions of the study by a request through an email to the corresponding author.
